# A retrospective comparative study of chronic subdural hematoma in patients with or without decreased level of consciousness

**DOI:** 10.1186/s12883-026-05207-7

**Published:** 2026-08-15

**Authors:** Xufei Guo, Li’ao Wang, Bingcheng Zhu, Yunfei Li, Weiming Liu, Liang Wu

**Affiliations:** 1 Department of Neurosurgery, Beijing Tiantan Hospital, Capital Medical University, Beijing, China; 2 China National Clinical Research Center for Neurological Diseases, Beijing, China; 3 Neurological Center, People’s Hospital of Ningxia Hui Autonomous Region (The Third Clinical Medical College, Ningxia Medical University), Yinchuan, China

**Keywords:** Chronic subdural hematoma, Disturbed consciousness, Complications, Modified Rankin scale, Risk factors, Coagulopathy

## Abstract

**Background:**

In this retrospective cross-sectional study, we aimed to investigate the effects of preoperative decreased level of consciousness (DOC) on 6-month outcome and the risk factors that compromised the consciousness at admission in patients with chronic subdural hematoma (CSDH).

**Methods:**

From August 2011 to August 2017, a total of 1075 patients were consecutively included and assigned into two groups based on consciousness assessment: 1035 cases with full consciousness and 40 cases with preoperative DOC. The Glasgow Coma Scale was used to assess the consciousness status. The clinical features, radiological findings, and clinical outcomes were compared between patients with and without preoperative DOC. A least absolute shrinkage and selection operator (LASSO) method was used for variable selection, and then the multivariate logistic regression was constructed.

**Results:**

With a median age of 66 years old, 182 (16.9%) females and 893 (83.1%) males were included. Preoperative coagulopathy (*p* = 0.003) and stroke (*p* = 0.022) were more frequently observed in patients with DOC after univariate analysis. Receiving comparable treatments, patients with DOC developed more postoperative pneumonia (*p* = 0.041) and worse 6-month modified Rankin Scale (mRS) (*p* = 0.004). LASSO found age, duration of symptoms, stroke, smoke, coagulopathy, and total hematoma volume appropriate to include in the final model. After multivariate logistic regression analysis, age above 70 (Odds Ratio 2.18, 95% CI 1.10–4.35, *p* = 0.025) and coagulopathy (Odds Ratio 2.89, 95% CI 1.52–5.62, *p* = 0.001) were associated with increased odds of developing preoperative DOC in CSDH patients.

**Conclusions:**

CSDH patients with preoperative DOC tended to have postoperative pneumonia and worse mRS at 6-month. The old age (age above 70) and preoperative coagulopathy might be associated with the compromised consciousness at admission. Identifying these associated factors early can assist neurosurgeons in clinical risk stratification and optimizing preoperative care to potentially mitigate complications.

## Introduction

Chronic subdural hematoma (CSDH) is a common neurosurgical condition characterized by progressive subdural collection formed by fibrinolysis of the hematoma’s membrane and fluid influx driven by the hematoma’s hyperosmolarity [1]. The development and progression of CSDH is a complex process. Edlmann et al. [2] propose that not only trauma, but also inflammation, angiogenesis, and fibrinolysis are involved in its pathogenesis. A burr hole irrigation followed by adequate drainage has been widely accepted as the gold standard treatment [1, 3, 4]. The entity was estimated to occur up to 79.4 per 100,000 people every year, and the elderly, especially people in their 80s, were increasingly affected due to the aging society on a worldwide scale [4–7]. CSDH seemed not a pure benign disease considering that the mortality within the population ranged from 0 to 32%, and morbidity from 0 to 25% [1, 8, 9]. A Japanese nationwide database analysis found a poor outcome impacted 28.4% patients, and more than half of patients aged over 70 presented with decreased level of consciousness (DOC) to some extent [4]. The preoperative DOC usually represents the severity of the entity and portends a potential worse recovery even after the standard surgical treatment [10].

Specifically, preoperative DOC often indicates severe brain compression, elevated intracranial pressure, or a higher state of metabolic brain stress. This vulnerable physiological state compromises the brain’s inherent recovery capacity, thereby predicting a worse clinical trajectory and a higher susceptibility to complications. Furthermore, identifying the specific risk factors that predispose patients to preoperative DOC is clinically vital. It allows neurosurgeons to recognize high-risk patients early upon admission, optimize modifiable factors such as coagulopathy before surgical intervention, and tailor intensive postoperative care to mitigate adverse outcomes. This study aimed to investigate the prognosis of CSDH patients who presented with preoperative impaired consciousness and to identify the independent risk factors for its occurrence through multivariate regression analysis.

## Methods

### Population and study parameters

**Fig. 1:**
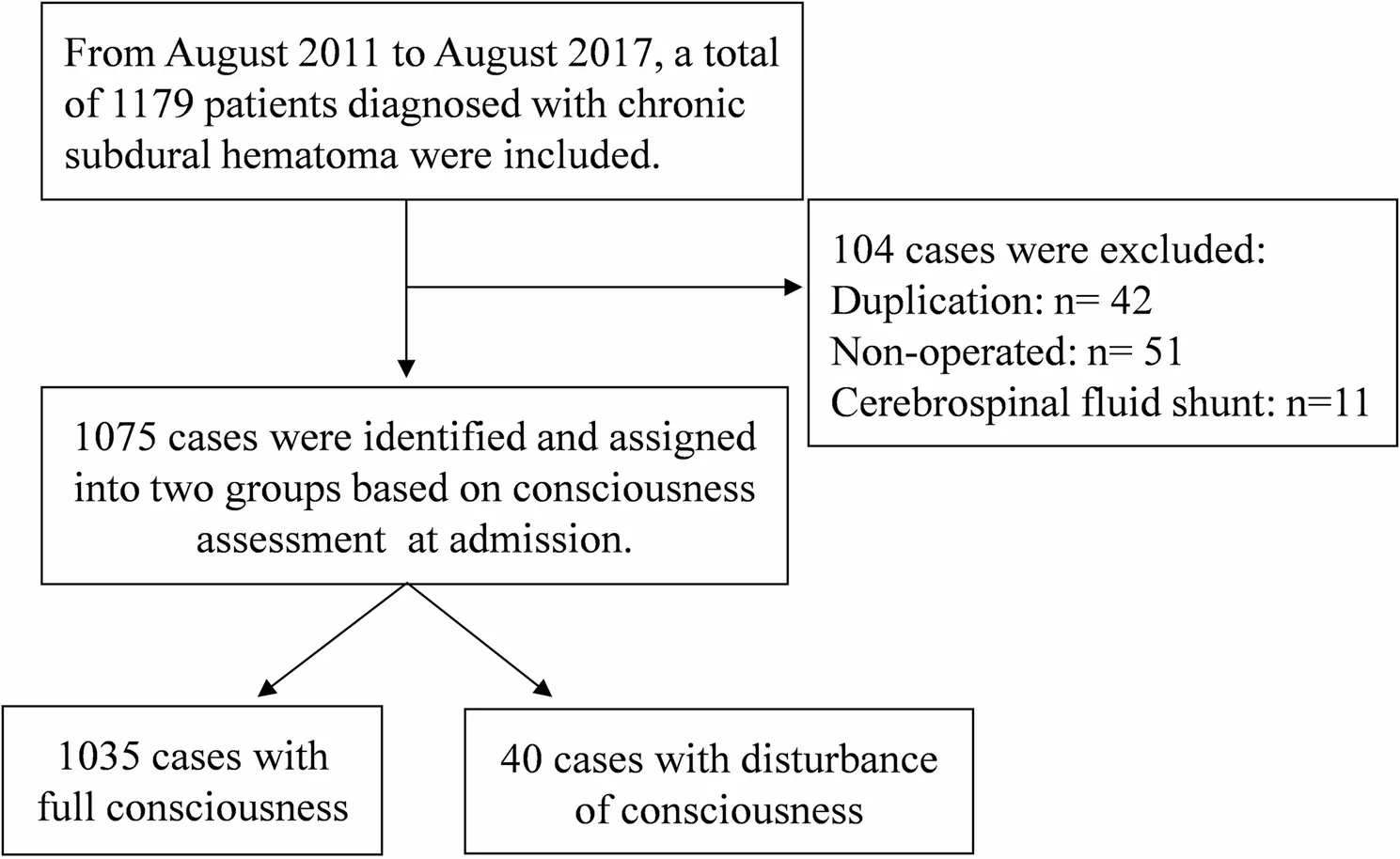
Flow chart of the study inclusion

From August 2011 to August 2017, a total of 1075 patients diagnosed with symptomatic CSDH were included (Fig. 1). Symptomatic CSDH was defined as a predominantly hypodense, isodense or mixed dense crescentic collection along the cerebral convexity on computed tomography (CT) scans [11]. Exclusion criteria were as follows: (1) patients aged < 18 years; (2) patients taking steroids or atorvastatin for CSDH, (3) patients refusing to undergo surgery, and (4) patients in the presence of cerebrospinal fluid shunt.

Clinical parameters such as demographics, location and volume of hematoma, complaints, pre-existing comorbidities, radiological classifications, interventions, and clinical outcomes were recorded consecutively. Decreased level of consciousness was defined as a state of reduced responsiveness to external stimuli—manifesting as confusion, lethargy, or coma—with a preoperative Glasgow Coma Scale (GCS) score of 14 or less [10, 12]. We used the Coniglobus formula (abc×1/2) to estimate the volume of subdural hematoma preoperatively [13]. Radiological presentations were classified as 7 subtypes, including hypo-, iso-, hyperdense, laminar, separated, gradation, and trabecular subtype [14]. Headache, limb weakness, dizziness, and dysphasia were mainly recorded, and the duration of symptoms was measured by days. We evaluated the coagulation profiles of all patients upon admission, including activated partial thromboplastin time (APTT), prothrombin time (PT), international normalized ratio (INR), thrombin time (TT), and fibrinogen levels. Any abnormality in these parameters was considered indicative of a preoperative coagulation disorder. Pre-existing comorbidities (hypertension, diabetes, coronary heart disease, stroke history, and subarachnoid cyst), anti-thrombotic drug use (aspirin and warfarin), and other variables (head trauma, alcohol, and cigarette use) were dichotomized. The burr hole procedure and drainage under local anesthesia were performed to all [3]. Urokinase was administered through draining catheter postoperatively when the subdural collection remained [3]. Complications such as pneumonia, seizure, fever, thrombotic events, and surgical site infection were reported. Deep venous thrombosis of the lower extremity, pulmonary embolus, heart attack, and ischemic stroke were defined as thrombotic events. Length of hospital stay was calculated as days from admission to discharge.

The modified Rankin scale (mRS) was applied to assess the clinical outcomes at 6 months after discharge, with scores of 0–2 indicating a good outcome and 3–6 representing a poor outcome [10, 15]. The assessments were determined by two neurosurgical residents through telephone or outpatient clinic visits using a standardized, structured mRS interview protocol to ensure assessment accuracy and minimize observer bias. Any disagreements regarding the mRS scores between the two residents were resolved through consensus or by consulting a third senior neurosurgeon to maintain high inter-observer agreement. We obtained ethics approval from the Institutional Research Ethics Committee (NO. KY 2020-094-02). Written informed consent was obtained from all enrolled patients.

### Statistical analyses

The dichotomous variables were presented by number and percentage, and the continuous ones were shown in median and interquartile range after being tested for normality. The baseline data were compared using Mann-Whitney U test, Pearson Chi-squared test, or Fisher’s exact test, respectively. For variable selection, we used univariate logistic regression firstly and set the criteria bar as p-value less than 0.2. Then a least absolute shrinkage and selection operator (LASSO) logistic regression algorithm was applied for further selection in the dataset. Variables with nonzero coefficients were thereby included. Lastly, a multivariate logistic regression model was constructed to investigate the potential associations. The result was presented in the forest plot, and p-values, odd radio (OR) and 95% confidence interval (CI) were shown (Fig. 3). P-value < 0.05 was defined statistically significant. Statistical analyses and graph-plotting were performed using R (version 4.1.1; R Development Core Team).

## Results

### Baseline characteristics

In this single-institute retrospective study, we included 1075 patients with CSDH, among which 40 patients suffered from DOC (Fig. 1). Table 1 shows detailed baseline clinical features of patients with chronic subdural hematoma with or without disturbed consciousness. The overall age distribution was negatively skewed. Age at diagnosis in the DOC group appeared higher than that in the full consciousness (FC) group. However, this comparison showed no statistical significance under Mann-Whitney U test (*p* = 0.085). The male to female ratio of CSDH was 4.9:1 in our dataset, and this gender ratio was similar in both FC and DOC groups (*p* = 0.458).

**Table 1:** Clinical features of chronic subdural hematoma patient with or without disturbed consciousness

Characteristics	Overall (*N*=1075)	Consciousness		*p*-value
		Full (*N*=1035)	Disturbed (*N*=40)	
Age (IQR), yr	66 (55, 76)	65 (55, 76)	72 (60.2, 78.2)	0.085^a^
Female (%)	182 (16.9%)	173 (16.7%)	9 (22.5%)	0.458^b^
Head Trauma (%)	724 (67.3%)	696 (67.2%)	28 (70.0%)	0.847^b^
Duration of syx, days	7 (6-9)	7 (6, 9)	7 (6, 12.2)	0.211^a^
Symptoms (%)				
Headache	640 (59.5%)	628 (60.7%)	12 (30.0%)	**<0.001** ^**b**^
Limb weakness	597 (55.5%)	574 (55.5%)	23 (57.5%)	0.926^b^
Dizziness	304 (28.3%)	299 (28.9%)	5 (12.5%)	**0.038** ^**b**^
Dysphasia	106 (9.9%)	101 (9.8%)	5 (12.5%)	0.585^c^
Coagulopathy (%)	367 (34.1%)	344 (33.2%)	23 (57.5%)	**0.003** ^**b**^
Aspirin (%)	112 (10.4%)	106 (10.2%)	6 (15.0%)	0.297^c^
Warfarin (%)	10 (0.9%)	10 (1.0%)	0 (0%)	1^c^
Smoke (%)	287 (26.7%)	281 (27.1%)	6 (15.0%)	0.128^b^
Alcohol (%)	209 (19.4%)	203 (19.6%)	6 (15.0%)	0.603^b^
Hypertension (%)	381 (35.4%)	370 (35.7%)	11 (27.5%)	0.367^b^
Diabetes (%)	206 (19.2%)	196 (18.9%)	10 (25.0%)	0.453^b^
CHD (%)	29 (2.7%)	27 (2.6%)	2 (5.0%)	0.294^c^
Stroke (%)	130 (12.1%)	120 (11.6%)	10 (25.0%)	**0.022** ^**c**^
Subarachnoid Cyst (%)	96 (8.9%)	92 (8.9%)	4 (10.0%)	0.776^c^
Bilateral (%)	299 (27.8%)	289 (27.9%)	10 (25.0%)	0.822^b^
Left (%)	740 (68.8%)	712 (68.8%)	28 (70.0%)	1 ^b^
Right (%)	634 (59.0%)	612 (59.1%)	22 (55.0%)	0.721^b^
Total Volume (IQR), ml	100 (77.36, 118.3)	100 (77.4, 118)	91 (68.3, 118)	0.227^a^
CT subtypes (%)				
Hypo	148 (13.8%)	136 (13.1%)	12 (30.0%)	**0.005** ^**b**^
Iso	319 (29.7%)	307 (29.7%)	12 (30.0%)	1^b^
Hyper	231 (21.5%)	225 (21.7%)	6 (15.0%)	0.411^b^
Laminar	85 (7.9%)	83 (8.0%)	2 (5.0%)	0.764^c^
Separated	50 (4.7%)	47 (4.5%)	3 (7.5%)	0.427^c^
Gradation	46 (4.3%)	44 (4.3%)	2 (5.0%)	0.687^c^
Trabecular	196 (18.2%)	193 (18.6%)	3 (7.5%)	0.114^b^

*CHD* Coronary Heart Disease, *CT* Computed Tomography, *IQR* Interquartile range, *syx* symptoms

^a^ Mann-Whitney U test, ^b^ Pearson Chi-square test; ^c^ Fisher’s exact test. Bold values indicate statistical significance ( *p* < 0.05)

Among all patients with symptomatic CSDH, 67.3% had head trauma, and the percentage of the event was comparable between two groups (*p* = 0.847). Patients with DOC complained of less headache (*p* < 0.001) and dizziness (*p* = 0.038). In addition, no statistical significance was observed in two groups concerning limb weakness (*p* = 0.926), verbal impairment (*p* = 0.585), and the duration of symptoms (*p* = 0.211). The coagulation panel at admission revealed abnormality in 57.5% patients with DOC whereas the percentage was 33.2% in FC group (*p* = 0.003). Preoperative aspirin use was recorded in 10.4% patients and warfarin use in 0.9%. We found the use of anti-thrombotic drugs was comparable between DOC and FC groups. In terms of personal history, we observed no difference in smoking (*p* = 0.128) and drinking habits (*p* = 0.603) between two groups. The pre-existing comorbidities showed 25% of patients with DOC had a stroke history, which affected more patients than in the FC group (11.6%, *p* = 0.022). In contrast, there was no statistical difference concerning hypertension, diabetes, coronary heart disease, and subarachnoid cyst (Table 1).

The median volume of the total hematoma was 100 ml, and the volume was comparable in both DOC and FC groups using Mann-Whitney U test (*p* = 0.227). CSDH occurred in the left convexity for 68.8%, right for 59%, and bilateral for 27.8%. The constituents were similar in patients with or without DOC. CSDH exhibited homogenous density (65%) in three subtypes, and they were hypo- (13.8%), iso- (29.7%), and hyperdense (21.5%). In addition, laminar (7.9%), separated (4.7%), gradation (4.3%), and trabecular (18.2%) types could also be observed in certain proportions. Specially, CSDH with homogenously hypodense imaging occurred in 30% patients with DOC versus 13.1% with full consciousness (*p* = 0.005).

### Clinical outcomes

We summarized the interventions and outcomes for patients with chronic subdural hematoma in Table 2. The burr hole procedure was performed for bilateral subdural hematoma in 19.4% cases, left in 45.2%, and right in 35.3%. The selection of surgical sites (*p* = 0.769) and the administration of urokinase (*p* = 0.670) postoperatively were both comparable between DOC and FC groups. Fever was the most frequent complication (2.8%), followed by pneumonia (0.8%), seizure (0.7%), thrombotic events (0.5%), and surgical site infection (0.3%). Pneumonia occurred more frequently in patients with DOC than in those without (*p* = 0.041). The median length of hospital stay was seven days in both groups. We assessed the patient statuses at six months postoperatively, and the constituents of mRS between DOC and FC groups were different (*p* = 0.003). In addition, the medians of mRS in the two groups also showed statistical difference (*p* = 0.004). We hereby stratified the distribution of mRS by good (mRS 0–2) and poor outcomes (3–6) and found the patient statuses at 6 months were worse in DOC group (*p* = 0.001).

**Table 2:** Intervention and outcomes of chronic subdural hematoma patient with or without disturbed consciousness

Variables	Overall (*N*=1075)	Consciousness		*p*-value
		Full (*N*=1035)	Disturbed (*N*=40)	
Burr hole (%)				0.769^a^
Left	486 (45.2%)	467 (45.1%)	19 (47.5%)	0.893^a^
Right	380 (35.3%)	365 (35.3%)	15 (37.5%)	0.903^a^
Bilateral	209 (19.4%)	203 (19.6%)	6 (15.0%)	0.603^a^
Frequency of Urokinase (IQR)	1 (0, 1)	1 (0, 1)	0.5 (0, 1)	0.670^b^
Complications (%)				
Pneumonia	9 (0.8%)	7 (0.7%)	2 (5.0%)	**0.041** ^**c**^
Seizure	8 (0.7%)	8 (0.8%)	0 (0%)	1^c^
Fever	30 (2.8%)	28 (2.7%)	2 (5.0%)	0.308^c^
Thrombotic events	5 (0.5%)	5 (0.5%)	0 (0%)	1^c^
Surgical Site Infection	3 (0.3%)	3 (0.3%)	0 (0%)	1^c^
Hospital stay (IQR), days	7 (6, 9)	7 (6, 9)	7 (6, 10.5)	0.395^b^
mRS at 6mos after surgery (%)				**0.003** ^**c**^
0	888 (82.6%)	861 (83.2%)	27 (67.5%)	
1	60 (5.6%)	59 (5.7%)	1 (2.5%)	
2	65 (6.0%)	61 (5.9%)	4 (10.0%)	
3	39 (3.6%)	35 (3.4%)	4 (10.0%)	
4	8 (0.7%)	6 (0.6%)	2 (5.0%)	
5	0 (0%)	0 (0%)	0 (0%)	
6	15 (1.4%)	13 (1.3%)	2 (5.0%)	
mRS at 6mos after surgery (IQR)	0 (0, 0)	0 (0, 0)	0 (0, 2)	**0.004** ^**b**^
Outcome at 6mos after surgery				**0.001** ^**c**^
Good (mRS≤2)	1013 (94.2%)	981 (94.8%)	32 (80.0%)	
Poor (mRS>2)	62 (5.8%)	54 (5.2%)	8 (20.0%)	

*IQR* interquartile range, *mRS* Modified Rankin Scale

^a^ Pearson Chi-square test; ^b^ Mann-Whitney U test; ^c^ Fisher exact’s test. Bold values indicate statistical significance ( *p* < 0.05)

### Risk factors

**Fig. 2:**
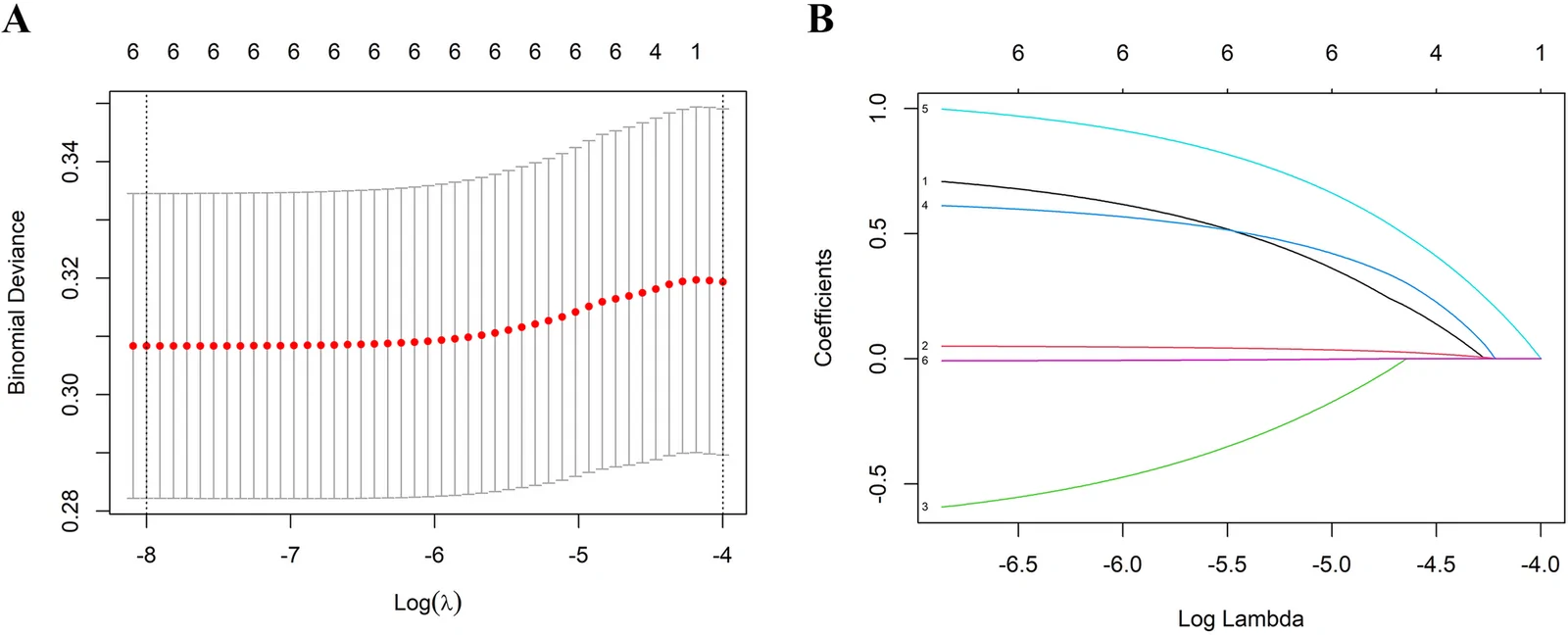
Variable selection using the LASSO logistic regression algorithm by 10-fold cross-validation. **A** A coefficient profile plot was generated against the log (lambda) sequence. **B** LASSO coefficients of six significant variables

**Fig. 3:**
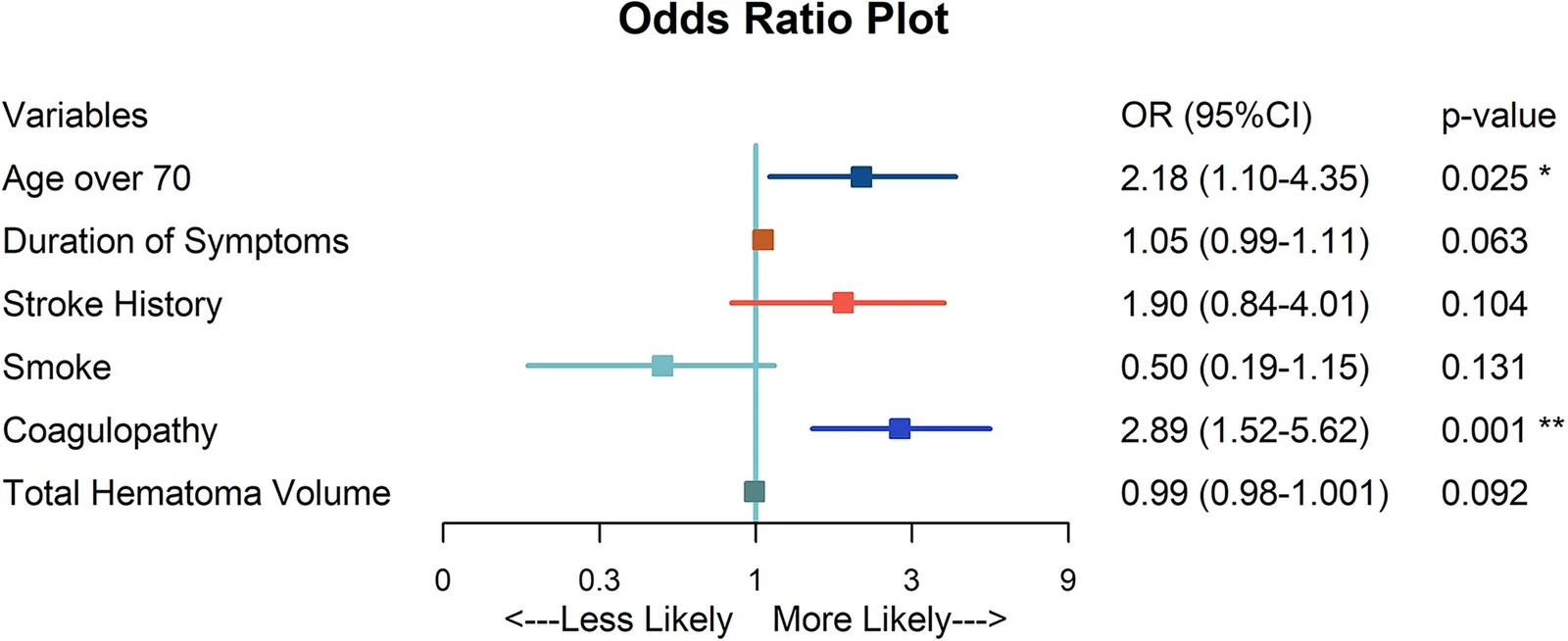
Forest plot of the results using the multivariate logistic regression model

Univariate logistic regression screening (using a p-value threshold of 0.2) revealed six candidate variables: age > 70 years, symptom duration, smoke, stroke history, coagulopathy, and total hematoma volume. These six features were considered potential options. We then used LASSO logistic regression algorithm to screen further for ideal variables, and we found the most appropriate tuning parameter λ was 0.00034 after cross-validation analysis (Fig. 2). All six variables were retained and thus included in the final model. After multivariate logistic regression analysis, the odds of developing preoperative DOC in aged group (age above 70) were 2.18 times the odds in younger group (age ≤ 70) (OR 2.18, 95%CI 1.10–4.35, p-value = 0.025). In addition, the odds of being affected by DOC were increased by 189% when the patients had coagulopathy at admission (OR 2.89, 95%CI 1.52–5.62, p-value = 0.001) (Fig. 3). We found no statistical significance of altered odds in duration of symptoms (OR 1.05, 95%CI 0.99–1.11, p-value = 0.063), stroke history (OR 1.90, 95%CI 0.84–4.01, p-value = 0.104), smoking (OR 0.50, 95%CI 0.19–1.15, p-value = 0.131), or total hematoma volume (OR 0.99, 95%CI 0.98–1.001, *p*-value = 0.092).

## Discussion

Multifactorial mechanisms associated with the development and progression of CSDH have been identified, and the monitoring and management of the certain variables could assist in the hospital care and better prognosis [2]. Especially, a level of preoperative consciousness could be quantified and clinically relevant to outcomes [16–20]. We thereby identified patients with disturbed consciousness based on preoperative GCS and found they accounted for 3.7% of our sample. We believe they correspond to a group of patients who may require special care. An epidemiological study of 63,358 CSDH patients in Japan reported more than half of patients aged 70 and above suffered DOC to some degree, which again necessitated the further exploration of the population. Our retrospective comparative study was based on a previous CSDH dataset [21], aiming to investigate the effects of preoperative DOC on 6-month outcome and the risk factors that compromised the consciousness at admission.

### Baseline characteristics and clinical outcomes

Our univariate analysis did not show a statistically significant age difference between patients with and without DOC. However, we found the old age (above 70) added odds of developing DOC at admission after adjustment, which was consistent with another study [4]. Previous studies on gender differences have suggested that females are less frequently affected by CSDH but experience more complications than males [22]. A study pointed out that the female sex was identified as an independent factor of DOC at admission and demonstrated the variable to be a death predictor at discharge [12]. Though the result was given through a multivariate analysis, their sample size was small. However, our findings following univariate and LASSO analyses contradicted this conclusion; therefore, the gender variable was excluded from our final model. We noticed headache and dizziness were less reported in patients with DOC and attributed this to a compromised verbal response.

Coagulation abnormalities were a critical factor which might be involved in the whole natural course of CSDH [16, 23, 24]. We compared the proportion of coexistent coagulopathy and found a significant difference between patients with and without DOC. Coagulopathy seemed to be associated with DOC in the population. Antithrombotic drug use, especially aspirin (10.4%), was common in our sample. A systematic review concluded that the antiplatelet medication was not correlated with increasing the rebleeding risk [25]. Another multicenter study supported the conclusion and added the antiplatelet use did not increase the persistent or worse functional impairment [26]. This might have explained why aspirin use was not directly associated with preoperative DOC in our study. As with subjective complaints, the assessment of smoking and drinking history was potentially limited by the patients’ impaired verbal responses in the DOC group, which might have potentially biased the weighing of other covariates in the model. A study focused on preexisting comorbidities pointed stroke might correlate to preoperative DOC and thereby influence the 90-day outcomes [10]. We agreed with the authors and found the significant difference between groups with and without DOC using Fisher’s exact test. However, the difference turned out not considerable after adjustment. We ascribed this result to a potential bias of the inclusion of smoking. The association between CT findings and level of consciousness in CSDH patients was researched by some investigators, and they found hyperdense hematoma would increase the severity of consciousness impairment [27]. Interestingly, we found patients with DOC tended to present with hypodense hematoma on CT scan, which was inconsistent with theirs. On one hand, those authors divided hematomas into only three categories (hypo-, iso-, and hyperdense), without accounting for the classic 7-subtype classification [14]. On the other, the stages of hypodense hematoma in the natural course of CSDH may vary. Some might consider hypodensity an earlier stage, while others regarded it as a sign of resolving stage [14, 28].

All treatments including surgery and urokinase administration were comparable between two groups. It was not unexpected that the outcomes of the two groups varied considerably [10, 16, 17]. The mRS at 6-month was measured and compared in the format of discrete and proportional data, and we found preoperative DOC affected outcomes at 6-month. Though there was no significant difference in the length of hospital stay, the postoperative pneumonia occurred more frequently in patients with preoperative DOC (*p* = 0.041). In addition to frailty before surgery, postoperative pneumonia might be another reason why those patients’ poor conditions endured [10].

### Age and preoperative decreased level of consciousness

Aging has been implicated in the chronic course of CSDH, influencing the development and prognosis [4, 7, 29]. However, little was known whether age was directly associated with preoperative DOC. After multivariate analysis, we found 70 was a cut-off value and the odds of patients aged above 70 developing DOC were 2.18 times higher (*p* = 0.025). A similar conclusion was drawn based on a large sample (63,358 CSDH patients) that DOC affected more than half of the patients aged above 70 [4]. In addition, about 40% of patients aged above 90 could not be discharged to home and the elderly patients exhibited a higher mRS at discharge [4]. All indicated that aging was inevitably involved in the severity of CSDH. Another investigator found patients aged above 75 tended to present with brain herniation (Odds ratio 2.16, *p* < 0.0001), which usually portended a dismal prognosis [19]. The authors attributed the delay in diagnosis to a focus on comorbidities and the progressively deteriorating consciousness amongst the older [19]. We know aging is a complex and pivotal process in a multifaceted fashion, involving increased inflammatory responses, impaired angiogenesis, and insufficient repair capacity, which happen to be deeply implicated in the CSDH [29]. It’s thereby reasonable that the old age (age above 70 in our study) might aggravate the preoperative consciousness status in an intricate pattern.

### Coagulopathy and preoperative decreased level of consciousness

New insights into the pathophysiology of CSDH are gaining wide acceptance, which put coagulation, angiogenesis, and inflammation at the core of CSDH formation [2, 29]. We found preoperative coagulopathy occurred to 34.1% CSDH patients and affected patients with DOC more frequently (Odds Ratio 2.89, *p* = 0.001). This was supported by another study, which found the international normalized ratio was significantly correlated with a worse GCS grading at admission [23]. A paucity of studies researched on the effects coagulopathy had on CSDH patients at admission, whereas most of them focused on the worse outcome coagulopathy caused [23, 24]. Our multivariate analysis indicated that coagulopathy might have played a detrimental role before surgery already, especially regarding preoperative level of consciousness. Consistent with our findings, Liebert et al. [30] similarly observed a significantly elevated preoperative INR in DOC patients versus controls (*p* = 0.004). Coagulopathy might be a modifiable and treatable factor if we figured out the causes [24, 31, 32]. We previously reported the use of antiplatelet was associated with preoperative coagulopathy [31]. In addition, other researchers pointed Factor XIII deficiency or liver diseases might be correlated [24, 32]. Further investigation into the causes might aid in the preoperative management of CSDH and help improve outcomes [24]. In short, we found coagulopathy might be a risk factor for DOC in CSDH, which requires monitoring and possible intervention once diagnosed.

Our findings offer several practical implications for the clinical management of CSDH. First, neurosurgeons should actively utilize admission DOC status and age (especially over 70) as key indicators for early risk stratification, recognizing that these patients are highly susceptible to postoperative pneumonia and poorer 6-month functional outcomes. Second, given the significant association between coagulopathy and preoperative DOC, we strongly recommend early and routine screening of coagulation profiles for all CSDH patients upon admission, particularly the elderly. Finally, these findings can directly optimize preoperative and postoperative planning. Identifying coagulopathy early allows for prompt medical correction prior to surgical intervention. Furthermore, anticipating the higher risk of postoperative pneumonia in patients presenting with DOC should prompt care teams to implement aggressive prophylactic pulmonary care and early mobilization in the postoperative period.

## Limitations

Our study has several limitations that should be acknowledged. First, as a retrospective observational study utilizing a single-center dataset, our findings are inherently susceptible to selection bias, which may limit the generalizability of the results to broader populations. Second, there is potential clinical heterogeneity in the definition and subjective assessment of DOC among different attending physicians, and the absence of adjustment for the patients’ preoperative functional baseline (such as a preoperative mRS) or routine cognitive assessments may introduce baseline imbalances, leaving some unmeasured confounders unconsciously excluded from our multivariate analysis. Third, regarding radiological parameters, the degree of midline shift—a critical variable known to heavily influence the level of consciousness—was not collected in our historical medical charts, and the total hematoma volume was estimated using the Coniglobus formula (abc/2) rather than being calculated by more precise modern 3D software reconstructions. Finally, due to the relatively small sample size of patients presenting with preoperative DOC (*n* = 40), we were unable to conduct robust subgroup analyses based on specific GCS score tiers or hematoma laterality (unilateral versus bilateral). We anticipate that future prospective, multicenter cohort studies with comprehensive data collection and 3D volumetric analyses will address these limitations.

## Conclusions

CSDH patients with preoperative DOC tended to have postoperative pneumonia and worse mRS at 6-month. The old age (age above 70) and preoperative coagulopathy were significantly associated with the compromised consciousness at admission. Recognizing these associations early can assist neurosurgeons in clinical risk stratification, allowing for the timely optimization of preoperative care and the implementation of tailored postoperative strategies to potentially mitigate adverse outcomes.

## Data Availability

The datasets used and/or analysed during the current study are available from the corresponding author on reasonable request.
